# A cross-sectional study of frequency and factors associated with dog walking in 9–10 year old children in Liverpool, UK

**DOI:** 10.1186/1471-2458-13-822

**Published:** 2013-09-10

**Authors:** Carri Westgarth, Lynne M Boddy, Gareth Stratton, Alexander J German, Rosalind M Gaskell, Karen P Coyne, Peter Bundred, Sandra McCune, Susan Dawson

**Affiliations:** 1 Institute of Infection and Global Health, Faculty of Health and Life Sciences, University of Liverpool, Leahurst Campus, Neston, Cheshire CH64 7TE, UK; 2 School of Veterinary Science, Faculty of Health and Life Sciences, University of Liverpool, Leahurst Campus, Neston, Cheshire CH64 7TE, UK; 3 Physical Activity, Exercise and Health Research Group, Research Institute for Sport and Exercise Sciences, Liverpool John Moores University, Liverpool L3 2ET, UK; 4 Sport and Health Portfolio, College of Engineering Swansea University, 942c Talbot Building, Singleton Park, Swansea SA2 8PP, UK; 5 Institute of Ageing & Chronic Disease, Faculty of Health and Life Sciences, University of Liverpool, Leahurst Campus, Neston, Cheshire CH64 7TE, UK; 6 Institute of Psychology Health and Society, Faculty of Health and Life Sciences, University of Liverpool, Whelan Building, Quadrangle, Brownlow Hill L69 3GB, UK; 7 WALTHAM® Centre for Pet Nutrition, Waltham-on-the-Wolds, Melton Mowbray, Leics LE14 4RT, UK

**Keywords:** Dog, Physical activity, Walking, Children, Attachment

## Abstract

**Background:**

Owning a pet dog could potentially improve child health through encouraging participation in physical activity, through dog walking. However, evidence to support this is limited and conflicting. In particular, little is known about children’s participation in dog walking and factors that may be associated with this. The objective of this study was to describe the participation of children in dog walking, including their own and those belonging to somebody else, and investigate factors associated with regular walking with their own pet dog.

**Methods:**

Primary school children (n=1021, 9–10 years) from a deprived area of Liverpool were surveyed during a ‘fitness fun day’ as part of the *Sports*Linx project. The ‘Child Lifestyle and Pets’ survey included questions about pet ownership, pet attachment, and dog walking. Multivariable logistic regression models were used to investigate factors associated with walking any dog, or their own dog, several times a day or more, including level of attachment to the dog, dog type, and sociodemographic factors.

**Results:**

Overall, 15.4% of children reported walking with any dog (their own or belonging to a friend or family member) ≥ once daily, 14.1% several times a week, 27.6% ≤ once a week, and 42.8% never. Dog owning children (37.1% of the population) more often reported dog walking ‘several times a week or more’ (OR=12.30, 95% CI=8.10-18.69, P<0.001) compared to those without a dog, but were less likely to report other walking without a dog. The majority (59.3%) of dog owning children indicated that they usually walked their dog, with 34.6% reporting that they walked their dog ≥ once daily. Attachment score was highly associated with the child reporting walking their dog (lower score=higher attachment; OR=0.93, 95% CI=0.89-0.96, P<0.001). There was no evidence that gender, ethnicity, sibling status or deprivation score was associated with dog walking. Children that reported owning Pit Bulls were more likely to report friends walking with their dog than those owning non-Pit bull types (OR=10.01, 95% CI=1.52-65.76, P=0.02, respectively).

**Conclusions:**

Promotion of supervised walking of suitable pet dogs may be an opportunity for increasing physical activity in 9–10 year old children. The identification of stronger attachment to dogs regularly walked is similar to findings in adult studies.

## Background

### Pets and health

Many adults and children own pets and are deeply attached to them. Pet ownership has been suggested to have positive effects on both physical and mental aspects of human health [1,2], but others argue that the evidence so far is inconclusive [3]. Much of the previous research has focused on pet owners facing significant health challenges, rather than studying the phenomenon of pet ownership in everyday life [4]. Despite many households owning pets, for example over half of households in the UK [5], the impact of pet ownership on households and families with children is not well understood [6].

### Dogs and physical activity

One proposed primary health benefit of regular contact with pet dogs is motivation for a more active lifestyle, through walking them [7]. The impact of dog ownership on physical activity is beginning to receive considerable research attention as a potential means of increasing total daily activity for a significant proportion of the population. It has been estimated that dog owning adults are 57% more likely than non-owners to achieve the recommended level of physical activity [8] and a number of studies (mainly from the USA and Australia) have now shown that greater physical activity can occur in adults through walking a pet dog [9]. Findings have also been confirmed through the use of objective measures such as accelerometry, and it has been suggested that dog walking can also be associated with lower weight status [10]. However, just because a person owns a dog, does not mean they necessarily walk it; for example a USA study reported 30% of owners did not walk their dog in the past week [10] and a recent survey by the UK Kennel Club estimated that at least a fifth did not walk every day [11]. The main contributing factors to regular compared to rare dog walking behaviour in adults appears to be owners that feel that their dog provides support and motivation to walk [12], a sense of ‘dog obligation’ [13] and report strong attachment to the dog [14], regardless of other potential factors such as socio-demographics.

### Dogs and physical activity in children

Pet dogs are more often found in households with middle-older age children [15,16] and, therefore, could be viewed as a vehicle for encouragement of an active lifestyle for a young population in the face of the obesity epidemic. In this regard, dogs are an accessible and arguably cost effective (as already present in many homes), prospective public health intervention for both children and adults [17]. However, even less information is available regarding the impact of dog ownership on the physical activity of children. An Australian study that examined 5–6 year old and 10–12 year old children suggested a positive association between dog ownership and objective measure of physical activity observed in girls only, and also with lower weight status in some groups studied [18,19]. One UK study suggested that children’s physical activity levels were marginally higher (357 steps per day) if they owned a dog, but did not examine concurrent associations with weight status or actual dog walking [20]. Another recent large UK population study suggested no evidence that children who own a dog are less likely to be obese [21]. A further Australian study again suggests that dog ownership in 10–12 yr olds is associated with increased activity but not lower weight status [22]. Thus, the evidence so far is that owning a pet dog does not strongly equate with a child being more physically active, nor having a lower weight status.

### Children’s participation in dog walking

This raises the question of how often children actually walk with their dogs and what may influence this. In one study, only 23% of 5–6 year old and 37% of 10–12 year old Australian children were reported to walk with their dog [19]. Another Australian study reported that for girls but not boys (mean age 13yrs), participation in dog walking was associated with a neighbourhood that was perceived as good for being physically active and concerns about safety of local roads [23]. Further studies, of the nature and timing of activities with the pet dog and in different social and geographic settings, are now required [20].

### Study aims

The current study used an established programme of child health and fitness monitoring in 9–10 year old primary school children in Liverpool, UK [24,25], to access and sample a cross-section of child participants about walking activities including with a dog, and including linkage to demographic information. The study aimed to:

1. Describe the frequency of child participation in dog walking (their own and dogs belonging to other friends and family members).

2. Investigate who (family members, friends) are reported to walk the household dog.

3. Compare the effect of dog ownership on child participation in dog walking and participation in walking without a dog. We hypothesised that some compensation may be found by choosing to be active with a dog over other physical activities.

4. Investigate factors that may be associated with reporting dog walking, including demographic factors, owning certain types of dogs (for example Bull Breeds), and the degree of ‘attachment’ that children have to their dog.

## Methods

### Data collection

The methods of data collection have been described previously [26]. Briefly, all primary schools within Liverpool Local Education Authority are invited to participate annually in the *Sports*Linx project [24,25]. Participation is subsequent to granted informed parental consent and participant assent, and after the completion of medical screening forms. Ethical approval for the addition of the Child Lifestyle and Pets (CLAP) questionnaire to a sample of the 2010–2011 *Sports*Linx data collection was obtained from North West 3 Research Ethics Committee – Liverpool East. Over ten weekdays in Oct-Nov 2010, 1091 9–10 year old children, from 31 schools, were sampled during attendance at *Sports*Linx Fitness Fun Days in Wavertree, Liverpool, a relatively deprived area. Children were asked to complete the CLAP questionnaire as part of their rotation of activities. Two University of Liverpool employees (one being the first author) supervised data collection, all of whom were trained in assisting children to complete the questionnaire. When available, schoolteachers and other *Sports*Linx instructors also assisted. The full questionnaire can be requested from the authors and further information is available [26].

### Outcomes

For the purposes of this study, children were asked how often (Never, Once a week or less, Several times a week, Once a day or more) they: walked with a dog (yours or someone else’s); walked without a dog (for example to or from school); walked with your own dog (if currently has one), and who usually walked the dog (yes/no - mother, father, child, brothers, sisters, friends, other). The ordinal variables regarding walk frequency were then further categorised into the binary variable once a week or less, or several times a week or more.

### Predictive variables

Children were also asked about pets currently owned (living in the household that the child spends most of its time in). Demographic information was also collected, this included: number of siblings, whether the child was the youngest; and time spent playing sports or outdoor games (never, up to 1 hour, up to 2 hours, up to 3 hours, over 3 hours). The strength of the relationship between the child and their ‘favourite’ pet owned (often termed ‘attachment’) was measured using a validated series of 27 questions with a Likert-type scale; the CENSHARE Pet Attachment Scale [27]. Information on dog type (breed or indication of mixed breed) was also collected, whilst parental consent forms were used to collect other data on gender, ethnicity (White UK, Black UK, African, Indian, Pakistani, Bangladeshi, Chinese, Somali, mixed or ‘other ethnicity’) and home postcode.

Ethnicity data were further categorised into white or non-white. Children were categorised as being a single child (no siblings), youngest child, or neither single nor youngest. Index of Multiple Deprivation 2007 (IMD2007) score was calculated from home postcode. An overall attachment score was determined by totalling the individual question responses into a total CENSHARE Score after reverse scoring 4 items. Participation in sports or outdoor games on a weekday was categorised into up to 1 hour, or over 1 hour. Dog-owning children were categorised as having single or multiple dogs. Information on dog types owned were used to create binary variables for each child as living with a ‘Pit Bull’ (including pseudonyms such as ‘Irish Staff’ and ‘Red Nose’ and also including cross-breeds containing Pit Bull) or ‘Bull breeds’ (Pit Bulls, Staffordshire Bull Terrier, Bulldog (often the American Bulldog), or crosses of these; and in addition the one Presa Canario was included in this grouping due to the breed association with fighting). These categorisations were chosen as bull breed types were very common in this population.

### Data analysis

Data were initially analysed in MINITAB to compare outcomes related to frequency of dog walking and who walks the dog, with predictive variables: using chi-squared tests and binary logistic regression for categorical variables such as presence of dog or dog types; T-tests or Analysis of Variance and regression for the continuous variable of attachment score as it was normally distributed; and continuous variable of IMD2007 score was analysed using the Kruskal-Wallis test as the data were not normally distributed. Adjusted binary logistic regression models were then built for the outcomes ‘walking with any dog’, ‘walking without a dog’, ‘walking with own dog’, and ‘friend walks dog’ using multivariable modelling of fixed effects: gender; ethnicity; sibling status (single, youngest or neither); deprivation score; sport or outdoor games; and as appropriate, dog ownership, single or multiple dogs, dog type and attachment score. Models were also tested in MLwiN with the additional hierarchical structure of ‘School’ as a random effect; there were negligible differences in the estimates and so standard models are presented here.

## Results

CLAP questionnaire data were available for 1021 children (94%). Some schools opted to keep the consent forms on their premises and, therefore, complete demographic data were available for 88.6% of the total intended sample (n=967). Demographic and descriptive pet ownership information has been reported elsewhere [26].

### Frequency and factors associated with walking with any dog or walking without a dog

Overall, 142 (15.4%) children reported walking with any dog (their own or belonging to a friend or family member) once a day or more, 130 (14.1%) several times a week, 254 (27.6%) once a week or less, and 394 (42.8%) never. This compares to walking for other purposes (without a dog) where 259 (28.7%) reported walking once a day or more, 228 (25.2%) several times a week, 196 (21.7%) once a week or less and 221 (24.5%) never.

Unsurprisingly, children were far more likely to report walking with any dog ‘several times a week or more’ if they had a dog compared to without (Table 1a. OR=12.30, 95% CI=8.10-18.69, P<0.001) but dog-owning children were less likely to report other walking (without a dog) than those without a dog (Table 1b. OR=0.58, 95% CI=0.42-0.81, P=0.001). There was no evidence of a difference between boys and girls, as to how often they reported walking any dog (Table 1a). However, there was some evidence that girls were more likely than boys to report walking without a dog but this was marginal (Table 1b. OR=1.38, 95% CI=1.00-1.90, P=0.05). There was no evidence after adjustment that ethnicity, sibling status, deprivation score, or frequency of participating in sports or outdoor games during the week, was associated with frequency of walking any dog or walking without a dog.

**Table 1:** Logistic regression analyses of factors associated with frequency of walking any dog, and frequency of walking without a dog, several times a week or more

**Outcome (predictor variable)**	**Category**	**Once a week or less (n (%) or median (IQR))**	**Several times a week or more (n (%) or median (IQR))**	**Crude OR (95% CI)**	**Adjusted OR (95% CI)**
**a) Walking with any dog**					
Gender	Male	341 (74.3)	118 (25.7)		
	Female	271 (65.5)	143 (34.5)	**1.52**	1.43
				(1.14–2.04)	(0.97–2.12)
Ethnicity	White	420 (67.2)	205 (32.8)		
	Non-white	93 (81.6)	21 (18.4)	**0.46**	1.00
				(0.28–0.76)	(0.53–1.87)
Sibling status	Neither	321 (71.0)	131 (29.0)		
	Single	71 (67.6)	34 (32.4)	1.17	1.28
				(0.74–1.85)	(0.67–2.44)
	Youngest	230 (69.3)	102 (30.7)	1.09	1.00
				(0.80–1.48)	(0.65–1.52)
Deprivation score	Continuous	38.4 (39.3)	43.2 (41.2)	**1.01**	1.00
				(1.00–1.02)	(0.99–1.01)
Sports/outdoor games during weekday	<1 hour	236 (74.9)	79 (25.1)		
	≥1 hour	385 (67.9)	182 (32.1)	**1.41**	1.48
				(1.04–1.93)	(0.97–2.25)
Owns a dog	No	501 (89.3)	60 (10.7)		
	Yes	147 (41.0)	212 (59.9)	**12.04**	**12.30**
				(8.57–16.93)	(8.10–18.69)
**b) Walking without a dog**					
Gender	Male	219 (49.1)	227 (50.9)		
	Female	174 (42.4)	236 (57.6)	**1.31**	**1.38**
				(1.00–1.71)	(1.00–1.90)
Ethnicity	White	283 (46.1)	331 (53.9)		
	Non-white	48 (42.1)	66 (57.9)	1.18	1.23
				(0.78–1.76)	(0.77–1.97)
Sibling status	Neither	204 (45.6)	243 (54.4)		
	Single	41 (40.2)	61 (59.8)	1.25	1.10
				(0.81–1.93)	(0.66–1.83)
	Youngest	156 (48.0)	169 (52.0)	0.91	0.91
				(0.68–1.21)	(0.65–1.29)
Deprivation score	Continuous	41.2 (40.3)	39.4 (41.3)	1.00	1.00
				(0.99–1.01)	(1.00–1.01)
Sports/outdoor games during weekday	<1 hour	149 (47.9)	162 (52.1)		
	≥1 hour	245 (44.1)	311 (55.9)	1.17	1.15
				(0.88–1.54)	(0.83–1.61)
Owns a dog	No	227 (40.7)	331 (59.3)		
	Yes	190 (55.1)	155 (44.9)	**0.56**	**0.58**
				(0.43–0.73)	(0.42–0.81)

Bold=OR P<0.05.

a) n=655. Hosmer-Lemeshow=0.75. Adjusted model for gender, ethnicity, sibling status, deprivation score, sports/outdoor games during weekday, owns a dog.

b) n=646. Hosmer-Lemeshow=0.68. Adjusted model for gender, ethnicity, sibling status, deprivation score, sports/outdoor games during weekday, owns a dog.

### Frequency and factors associated with walking with own dog

Three-hundred and seventy-eight children (37.1%) reported owning a dog, ranging from one (n=283, 75.5%) to nine (n=2, 0.5%).

When asked ‘who usually walks with your dog?’, 217 (59.3%) of dog-owning children indicated that they usually walked with their dog, 157 (42.9%) indicated their mother, 163 (44.5%) indicated their father, 116 (31.7%) their brothers and sisters, 54 (14.8%) their friends, and 42 (11.4%) ‘other’ who included other family members such as grandparents, cousins and uncles, and step-family. One hundred and thirteen (34.6%) children reported walking with their dog once a day or more, 96 (29.4%) several times a week, 88 (26.9%) once a week or less, and 30 (9.2%) never.

Children who indicated that they usually walked with their dog reported lower CENSHARE total scores, and thus higher attachment to the dog (ANOVA P=0.002; means: Never=54.6, Once a week or less=53.1, Several times a week=45.9, Once a day or more=47.2).

There was some evidence that children who reported walking with their own dog, several times a week or more, were more likely to also report participating in sports and outdoor games, over 1 hour per weekday, than those who reported walking their dog once a week or less (OR=2.06, 95% CI=1.15-3.69, P=0.02, Table 2: Model 1). However, after addition of attachment score to the dog (in Table 2: Model 2) the significance of this association somewhat attenuated (P=0.05). Both models are presented here because Model 2 is based on substantially less data than Model 1 due to the availability of full CENSHARE attachment score data and the favourite pet being a dog. Table 2: Model 2 had better model fit than model one (Hosmer-Lemeshow 0.55 compared to 0.32) demonstrating that, even after adjustment, attachment score was still highly associated with the child reporting walking their own dog (lower score=higher attachment; OR=0.93, 95% CI=0.89-0.96, P<0.001). There was no evidence that gender, ethnicity, sibling status or deprivation score was associated with the frequency of child walking their own dog. There was also no direct evidence that having more dogs was associated with more dog walking; in fact, any effect direction of this was negative, although not significant.

**Table 2:** Logistic regression analyses of factors associated with child reporting that they walk with their own dog several times a week or more

**Outcome (predictor variable)**	**Category**	**Once a week or less (n (%) or median (IQR) or mean (SD))**	**Several times a week or more (n (%) or median (IQR) or mean (SD))**	**Crude OR (95% CI)**	**Model 1 Adjusted OR (95% CI)**	**Model 2 Adjusted OR (95% CI)**
**Freq walks with own dog**						
Gender	Male	50 (34.3)	96 (65.8)			
	Female	59 (36.4)	103 (63.6)	0.91	0.80	0.99
				(0.57–1.45)	(0.46–1.40)	(0.46–2.12)
Ethnicity	White	85 (34.3)	163 (65.7)			
	Non-white	7 (33.3)	14 (66.7)	1.04	1.94	0.46
				(0.41–2.68)	(0.61–6.14)	(0.10–2.11)
Sibling status	Neither	52 (34.2)	100 (65.8)			
	Single	12 (37.5)	20 (62.5)	0.87	0.73	0.49
				(0.39–1.91)	(0.28–1.91)	(0.11–2.27)
	Youngest	49 (37.1)	83 (62.9)	0.88	0.90	0.89
				(0.54–1.43)	(0.50–1.63)	(0.39–2.02)
Deprivation score	Continuous	50.0 (33.9)	48.8 (6.7)	1.00	0.99	1.00
				(0.98–1.01)	(0.98–1.01)	(0.98–1.02)
Sports/outdoor games during weekday	<1 hour	53 (47.8)	58 (52.3)			
	≥1 hour	58 (28.6)	145 (71.4)	**2.28**	**2.06**	**2.24**
				(1.41–3.70)	(1.15–3.69)	(0.99–5.11)
Number of dogs	Single	87 (35.7)	157 (64.3)			
	Multiple	31 (37.8)	51 (62.2)	0.91	0.88	0.45
				(0.54–1.53)	(0.46–1.68)	(0.18–1.12)
Bull Breed	No	88 (37.1)	149 (62.9)			
	Yes	30 (33.3)	60 (66.7)	1.18	1.39	1.65
				(0.71–1.97)	(0.72–2.67)	(0.63–4.33)
Attachment score	Continuous	53 (12.0)	47 (11.0)	0.95		**0.93**
				(0.92–0.98)		(0.89–0.96)

Bold=OR P<0.05.

Model 1: n=233. Hosmer-Lemeshow=0.32. Adjusted model for gender, ethnicity, sibling status, deprivation score, sports/outdoor games during weekday, number of dogs, bull breed.

Model 2: n=144. Hosmer-Lemeshow=0.55. Adjusted model for gender, ethnicity, sibling status, deprivation score, sports/outdoor games during weekday, number of dogs, bull breed, attachment score.

We also investigated factors associated with who is reported to walk the dog, and report findings for the child reporting that their friends assist them with walking their dog (Table 3); no associations were found for other family members. Non-whites were more likely to report friends walking their dog than white children (OR=7.47, 95% CI=2.50-22.27, P=<0.001, Table 3: Model 1); however, after inclusion of attachment (Model 2; but again a smaller dataset now), this association disappeared (P=0.22). In Model 2 there was also some evidence that children with multiple dogs were less likely to report their friends walking their dog than children with single dogs (OR=0.21, 95% CI=0.05-0.92, P=0.04). In Table 3: Model 3 a further model is presented that used Pit Bull as dog type instead of Bull Breeds. Interestingly, this model suggested that children owning Pit Bulls were more likely to report their friends walking with their dog than those owning non-Pit bull types (OR=10.01, 95% CI=1.52-65.76, P=0.02) and the relationship with ethnicity was no longer present. There was no evidence that gender, sibling status, deprivation score, participation in sports or outdoor games during the week, or attachment score, were associated with reporting friends walking with the dog.

**Table 3:** Logistic regression analyses of factors associated with child reporting that their friends usually walk with their dog

**Outcome (predictor variable)**	**Category**	**No (n (%) or median (IQR) or mean (SD))**	**Yes (n (%) or median (IQR) or mean (SD))**	**Crude OR (95% CI)**	**Model 1 Adjusted OR (95% CI)**	**Model 2 Adjusted OR (95% CI)**	**Model 3 Adjusted OR (95% CI)**
**Friend walks dog**							
Gender	Male	145 (86.8)	22 (13.2)				
	Female	150 (84.3)	28 (15.7)	1.23	1.00	0.44	0.47
				(0.67–2.25)	(0.50–2.01)	(0.16–1.17)	(0.17–1.26)
Ethnicity	White	237 (86.2)	38 (13.8)				
	Non-white	14 (60.9)	9 (39.1)	**4.01**	**7.47**	2.78	2.19
				(1.62–9.91)	(2.50–22.27)	(0.53–14.43)	(0.36–13.18)
Sibling status	Neither	140 (83.8)	27 (16.2)				
	Single	30 (83.3)	6 (16.7)	1.04	1.38	0.41	0.37
				(0.39–2.73)	(0.44–4.30)	(0.04–4.12)	(0.03–4.73)
	Youngest	129 (86.0)	21 (14.0)	0.84	0.91	2.06	2.22
				(0.45–1.57)	(0.43–1.92)	(0.74–5.76)	(0.78–6.30)
Deprivation score	Continuous	47.0 (37.5)	50.2 (33.4)	1.01	1.00	1.01	1.01
				(0.99-1.02)	(0.99–1.02)	(0.98–1.04)	(0.98–1.03)
Sports/outdoor games during weekday	<1 hour	112 (88.2)	15 (11.8)				
	≥1 hour	185 (83.0)	38 (17.0)	1.53	1.84	2.18	2.19
				(0.81–2.91)	(0.83–4.07)	(0.70–6.80)	(0.71–6.81)
Number of dogs	Single	232 (84.1)	44 (15.9)				
	Multiple	79 (88.8)	10 (11.2)	0.67	0.47	**0.21**	0.27
				(0.32–1.39)	(0.19–1.19)	(0.05–0.92)	(0.06–1.17)
Bull Breed	No	229 (85.8)	38 (14.2)				
	Yes	83 (83.8)	16 (16.2)	1.16	1.10	2.81	
				(0.62–2.19)	(0.50–2.41)	(0.95–8.32)	
Pit bull	No	296 (86.1)	48 (14.0)				
	Yes	16 (72.7)	6 (27.3)	2.31			**10.01**
				(0.86–6.20)			(1.52–65.76)
Attachment score	Continuous	50 (12.2)	47 (8.2)	0.98		0.97	0.97
				(0.94–1.02)		(0.92–1.02)	(0.92–1.02)

Bold=OR P<0.05.

Model 1: n=259. Hosmer-Lemeshow=0.26. Adjusted model for gender, ethnicity, sibling status, deprivation score, sports/outdoor games during weekday, number of dogs, bull breed.

Model 2: n=157. Hosmer-Lemeshow=0.44. Adjusted model for gender, ethnicity, sibling status, deprivation score, sports/outdoor games during weekday, number of dogs, bull breed, attachment score.

Model 3: n=157. Hosmer-Lemeshow=0.39. Adjusted model for gender, ethnicity, sibling status, deprivation score, sports/outdoor games during weekday, number of dogs, pit bull, attachment score.

## Discussion

Although the majority (~60%) of dog owning children in this study reported that they usually walked their dog, there was a substantial proportion (~40 %) who reported that they did not usually walk their dog. As previous research indicates that only 4% of the dogs were reported never to be walked [26], this indicates that other friends and family were performing this role without the child. Just over a third of the dog owning children reported that they walked their dog once a day or more, as would be the ideal case for health benefits. The findings of this study suggest that walking pet dogs is a useful but under-utilised opportunity for physical activity for 9-10 year old children in Liverpool, UK.

In this context, the figure of 9% that reported never walking with their own dog is small compared with 41% of children in an Australian study who were reported (by the parents) never to walk with their dog [18]. This suggests that the children in our study may have over-reported walking with the dog compared to the response that parents may have given if asked. Alternatively, it may be an accurate representation of the situation, perhaps reflecting cultural and geographic differences between the study populations.

Stronger attachment to the pet was highly associated with regular walking with the dog. This concurs with previous evidence that the main contributing factors to regular compared to rare dog walking behaviour in adults appears to be owners that feel that their dog provides support and motivation to walk [12], a sense of ‘dog obligation’ [13] and report strong attachment to the dog [14], regardless of other potential factors such as socio-demographics. It is not known whether having a stronger bond with a dog causes walking, or walking a dog strengthens the bond between dog and owner, but it is likely a bit of both. It is unlikely that our finding was simply due to the increased appropriateness of the one CENSHARE item concerning physical activity with the pet (spending time playing with or exercising a pet), as the difference in average scores was too large.

There was some evidence that reporting that friends also walk with their dog was seven times more likely if the child was non-white, ten times more likely if the child reported owning a Pit Bull type dog, but was a fifth less likely if the child owned more than one dog. These relationships are certainly not clear and require more investigation as they confound each other. However, they are interesting observations as Maher and Pierpoint (2011) have previously observed that so-called ‘status’ or ‘weapon’ dogs such as Pit Bulls play a role of companionship, socialisation and protection in youth gangs in deprived inner city areas [28]. We previously reported that non-white children were more likely to report owning a Pit Bull type (but not the broader category of Bull Breeds) than White children and speculated that this could be a reflection of actual ownership of preferred dog types by different ethnic groups, or it could be due to non-whites being less inhibited in reporting that their dog is a Pit Bull, due to social and cultural reasons [26]. Based upon these findings, we hypothesise that here may be an association between ethnicity, dog type owned and socialising in groups of friends with dogs, in children even at this young age.

We also found some evidence that children were twice as likely to report regular walking with their own dog if they also reported participation in sports or outdoor games of one hour a day or more. This may be due to the children viewing dog walking activities as inclusive in ‘sports and outdoor games’ when estimating the time spent in these activities. Alternatively, more active children may be more likely to enjoy participating both in sports and outdoor games, and walking with their dog. If our second hypothesis is taken to be true, the question remains as to whether dogs make children more active or more active children acquire pet dogs. It is known that active parents tend to raise active children [29] and parents are important role models [30] that can reinforce and support participation in physical activities [31]. Thus, it may be that more generally active families also choose to have dogs.

However, our findings also showed that, not surprisingly, children who owned a dog were (twelve times) more likely to report walking any dog regularly compared to those without a dog, but were half as likely to report walking without a dog several times a week or more, than children without dogs. Thus, interestingly, time spent walking a dog may be in part ‘instead of’ rather than ‘additional to’ other time spent walking without a dog. It has previously been observed in adults that once dog walking was removed from the equation, dog walkers walked less than those without a dog; this led to a similar conclusion, that dog owners are selecting to be active with their dogs [13].

The strengths of this study are that the sample of children was relatively large, not convenience-based, and had high response rates, due to the specific context of data collection where all children were captured for a time period set aside purely for this purpose, and enthused to complete the questionnaire with support freely available. We also used multivariable regression modelling, to adjust for the confounding effects that demographic variables can have on each other, methods often lacking from previous literature in the study of human-animal Interactions. Demographic variables such as age, gender, socioeconomics and ethnic status are known to be associated with many health behaviours [32], and ownership of different pet types in adults [15,16,33,34], and children [35], including this sample [26].

However, the data, like much human-animal interaction research, are limited by the nature of self-report [3], as we did not measure dog walking objectively, nor ask the parents for verification. We also did not have verification of pets owned or dog type. The data were collected from a specific population, 9–10 year old children attending primary schools in a notably deprived area of Liverpool, and thus may not be generalisable to other UK cities or country-wide, nor other age groups. Cross-sectional HAI observational studies such as ours are also limited in their ability to infer the direction of causation [3]. The study also did not include how wider environmental factors may influence dog walking activity, as many factors known to influence physical activity in general, such as neighbourhood walkability, park attractiveness and safety, are also thought to influence dog walking [10,23,36].

Future research should elaborate on the intensity of dog walking participation, for example total minutes per week spent walking a dog, and intensity of exercise during dog walking as measured by accelerometers. Investigation of activities and behaviours of both dog and child during walking would clarify those that promote the most health-enhancing types of physical activity. Concurrent measurements of dog walking in parents as well as children would elucidate whether children do not walk with the dog because the dog is not being walked at all, or because another family member is walking the dog without the children, and the influence that parental involvement in dog walking can have on child physical activity. Perceived barriers and motivators to participation of children in dog walking activities should be investigated, incorporating both qualitative and quantitative examination of the perceptions of children and parents surrounding the role of their pet dog and feelings about walking with it. In addition, longitudinal studies should be used to investigate the effect of acquisition of a dog on long-term involvement in dog walking by children and parents. Such research will inform the design of possible future interventions to promote children to be more active with a suitable pet dog, under parental supervision [17].

Finally, consideration must be made to the complexities of the effects of dog ownership on the health and wellbeing of society. Although evidence suggests that owning a dog can confer both physical and mental health benefits [1-3] it also poses risks to health, for example zoonoses, bites, noise pollution, and the stress of dealing with behavioural problems [37]. Concerns about dogs being walked may also be a barrier to physical activity in non-owners and thus efforts to promote dog ownership or dog walking must be planned carefully [38].

## Conclusions

In summary, just over half of children reported participating in walking of their family pet dogs but not generally on a regular basis. Promotion of supervised walking of suitable pet dogs may be an opportunity for increasing physical activity in 9–10 year old children. The identification of stronger attachment to dogs regularly walked is similar to findings in adult studies.

## Competing interests

We declare that: (1) CW, SD, RMG, PB, AJG, and KPC had institutional support via a grant from WALTHAM® and Mars Petcare UK both divisions of Mars Inc. for the submitted work; the position of CW was funded from this grant but she is now funded by the Medical Research Council Population Health Scientist Fellowship; (2) the position of AJG at the institution is funded by Royal Canin, a subsidiary of Mars Inc and he has also received other research grants from Mars Petcare; (3) SMcC is an employee (Research Manager) of WALTHAM®, a division of Mars Inc; (4) LMB and GS have no financial or personal relationships with other people or organisations that could inappropriately or influence or bias the content of the paper.

## Authors’ contributions

CW conceived and designed the study, collected the data, performed the data analysis and drafted the paper. LMB and GS provided access to data collection and advised on study design and data analysis. SD, PB, AJG, RMG, and KPC were involved in conception of the study, study design and interpretation of findings. AJG and KPC also assisted with data collection and SD was also principal investigator. SMcC assisted in study design and interpretation of findings. All authors read and approved the final manuscript.

## Pre-publication history

The pre-publication history for this paper can be accessed here:

http://www.biomedcentral.com/1471-2458/13/822/prepub
